# Activated Leukocyte Cell Adhesion Molecule Expression and Shedding in Thyroid Tumors

**DOI:** 10.1371/journal.pone.0017141

**Published:** 2011-02-22

**Authors:** Francesca Miccichè, Luca Da Riva, Marina Fabbi, Silvana Pilotti, Piera Mondellini, Silvano Ferrini, Silvana Canevari, Marco A. Pierotti, Italia Bongarzone

**Affiliations:** 1 Proteomics Laboratory, Department of Experimental Oncology and Molecular Medicine, Fondazione IRCCS Istituto Nazionale dei Tumori, Milan, Italy; 2 Laboratory of Immunological Therapy, Istituto Nazionale per la Ricerca sul Cancro, Genoa, Italy; 3 Laboratory of Experimental Molecular Pathology, Department of Pathology, Fondazione IRCCS Istituto Nazionale dei Tumori, Milan, Italy; 4 Unit of Molecular Therapies, Department of Experimental Oncology and Molecular Medicine, Fondazione IRCCS Istituto Nazionale dei Tumori, Milan, Italy; 5 Scientific Directorate, Fondazione IRCCS Istituto Nazionale dei Tumori, Milan, Italy; Institut National de la Santé et de la Recherche Médicale - Institut Cochin, France

## Abstract

Activated leukocyte cell adhesion molecule (ALCAM, CD166) is expressed in various tissues, cancers, and cancer-initiating cells. Alterations in expression of ALCAM have been reported in several human tumors, and cell adhesion functions have been proposed to explain its association with cancer. Here we documented high levels of ALCAM expression in human thyroid tumors and cell lines. Through proteomic characterization of ALCAM expression in the human papillary thyroid carcinoma cell line TPC-1, we identified the presence of a full-length membrane-associated isoform in cell lysate and of soluble ALCAM isoforms in conditioned medium. This finding is consistent with proteolytically shed ALCAM ectodomains. Nonspecific agents, such as phorbol myristate acetate (PMA) or ionomycin, provoked increased ectodomain shedding. Epidermal growth factor receptor stimulation also enhanced ALCAM secretion through an ADAM17/TACE-dependent pathway. ADAM17/TACE was expressed in the TPC-1 cell line, and ADAM17/TACE silencing by specific small interfering RNAs reduced ALCAM shedding. In addition, the CGS27023A inhibitor of ADAM17/TACE function reduced ALCAM release in a dose-dependent manner and inhibited cell migration in a wound-healing assay. We also provide evidence for the existence of novel O-glycosylated forms and of a novel 60-kDa soluble form of ALCAM, which is particularly abundant following cell stimulation by PMA. ALCAM expression in papillary and medullary thyroid cancer specimens and in the surrounding non-tumoral component was studied by western blot and immunohistochemistry, with results demonstrating that tumor cells overexpress ALCAM. These findings strongly suggest the possibility that ALCAM may have an important role in thyroid tumor biology.

## Introduction

Thyroid tumours are the most frequent malignancies of the endocrine system [1]. The most common type is papillary thyroid carcinoma (PTC), a well-differentiated tumor arising in follicular cells that accounts for 80–90% of all thyroid malignancies. Medullary thyroid cancer (MTC) accounts for 5 to 10 percent of thyroid cancer cases and arises from calcitonin-producing C cells [2].

The epithelial to mesenchymal transition (EMT) is an essential step for invasiveness and progression in these tumors [3]; [4]. Crucial to this transition is the downregulation of cell–cell contacts, most notably E-cadherin–based adhesion [5]. Indeed, adhesion pathways and their altered regulation by B-catenin and Wnt signaling are important in the progression of thyroid tumors [6].

Among cell adhesion molecules, the neuron–glia-related cell-adhesion molecule (NrCAM) has been shown to be involved in thyroid carcinogenesis [7]–[9]. The loss of neural cell adhesion molecule CD56/NCAM expression is significant in papillary carcinoma (up to 100%), and such loss can serve as a specific and sensitive marker of PTC [10]; [11]. This gradual change seems to parallel a decrease in nuclear expression of thyroid transcription factor (TTF-1), an epithelial-specific transcription factor which regulates the changes in gene expression patterns that underlie EMT [12].

This report focuses on the activated leukocyte cell adhesion molecule (ALCAM or CD166), a member of the immunoglobulin superfamily, [13] in papillary and medullary thyroid tumors. Altered expression of ALCAM has been associated with differentiation state and progression in many tumors [14]–[21]. In addition, ALCAM is a marker of cancer stem cells and its expression at the tumor cell surface has been correlated with shortened survival in colon-rectal cancers [17]; [22] and with the vertical growth phase of progression in cutaneous melanoma [23].

On the contrary, in breast [24]–[26] and ovarian cancer [20] ALCAM cytoplasmic overexpression and low membrane expression were associated with disease progression. Therefore ALCAM at the cell surface plays a divergent role in the progression of different tumor types. Very recent data indicate that both transfection of mimics of microRNA-192 or -215 or ALCAM-specific siRNA significantly inhibit ALCAM expression and increase migration in a cell line model of gastric cancer [27]. Collectively these data suggest that the ALCAM relocalization from the cell membrane to cytoplasm might ultimately enhance the migratory properties of malignant cells facilitating metastatic dissemination in several cancers.

In this context, we have previously demonstrated that ALCAM is released from epithelial ovarian cancer (EOC) cells by a metalloprotease-dependent mechanism, leading to the generation of a natural soluble form of ALCAM that contains the greater part of the ectodomain. We have also shown that ALCAM shedding from the EOC can be enhanced by stimuli such as pervanadate (PV), phorbol myristate acetate (PMA), and epidermal growth factor (EGF) and can be blocked by inhibitors of ADAMs and by ADAM17/TACE silencing [28]. The clinical relationship of membrane ALCAM loss with progression in ovarian and breast cancer may also relate to the process of ALCAM shedding by ADAM17/TACE. Indeed, ADAM17/TACE inhibitors reduced ovarian carcinoma cell motility in vitro [28]. In addition, shedding of ALCAM results in the increase of sALCAM in the serum of ovarian [28] and breast cancer patients [21], suggesting a potential role of sALCAM as tumor biomarker, although lower levels are also present in the plasma of healthy individuals [29]. Besides enzymatic shedding, a recent report indicates that DNA-methylation of ALCAM promoter might also be involved in surface ALCAM down-regulation and breast cancer metastases development [24].

In view of the role of ALCAM dynamics in some human tumors, we studied its expression in thyroid tumors, which had not been previously explored. We also specifically investigated whether ADAM17/TACE was responsible for shedding of ALCAM in thyroid cancer cells and we found the existence of a novel 60-kDa soluble form of ALCAM. Finally, we studied the regulation of ADAM17/TACE activity by PMA and EGF stimulation, and the effects of ADAM17/TACE blockade in migration and invasion.

## Results

### TPC-1 cell line expresses ALCAM isoforms

**Figure 1:**
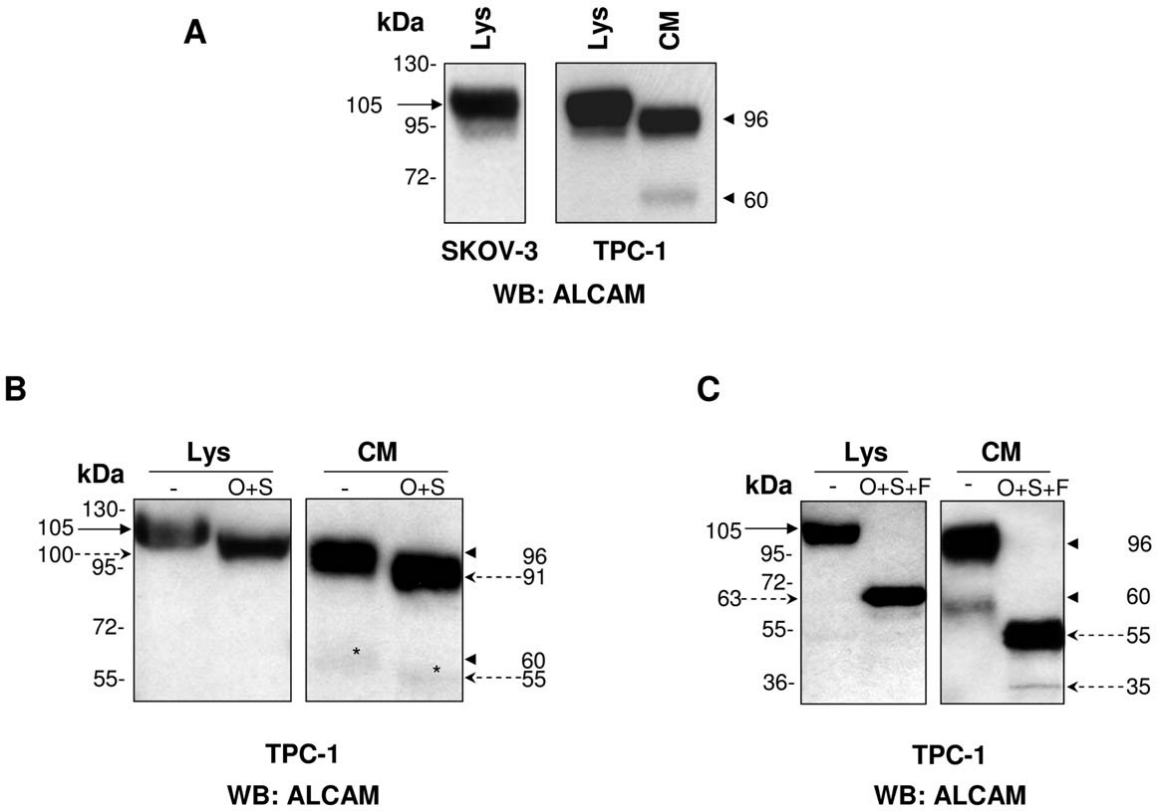
Western blot analysis of ALCAM expression in lysate and conditioned medium of TPC-1 cell line. (A) Analysis of TPC-1 cell lysate (Lys) and conditioned medium (CM) using anti-ALCAM antibody. Arrow indicates membrane-localized ALCAM isoform in lysate, and arrowheads indicate shed-ALCAM isoforms in CM, respectively. The SKOV-3 cell line was used as positive control. (B) TPC-1 cell lysate and CM were treated or not with a mixture of O-glycosidase (O) and sialidase (S) and analyzed by western blot using anti-ALCAM antibody. Continuous arrows indicate membrane and shed ALCAM forms before digestion, and dashed arrows indicate digestion products. (*) indicates less evident bands of ALCAM in CM. (C) TPC-1 cell lysate and CM were treated or not with O-glycosidase (O), sialidase (S), or PNGaseF (F) and analyzed by western blot using anti-ALCAM antibodies. Continuous arrows indicate membrane and shed ALCAM forms before digestion, and dashed arrows indicate digestion products.

Western blot analysis revealed the expression pattern of ALCAM in total cell lysate and in conditioned medium of the TPC-1 cell line (Fig. 1A). A single band of 105 kDa, corresponding to the fully glycosylated isoform in the cell lysate and two distinct isoforms of 96 and 60 kDa, respectively, were detected in conditioned medium. These results were consistent with those obtained with the SKOV-3 cell line known to express high levels of ALCAM (Fig. 1A). We then hypothesized that the 96-kDa isoform could correspond to a natural soluble form of ALCAM, which contains the greater part of the ectodomain, [28] while the 60-kDa isoform could correspond to a new isoform resulting from an alternative cleavage or differential glycosylation.

The glycosylation patterns of the ALCAM isoforms in cell lysates and conditioned medium were characterized by treatment with a mixture of endo-O-glycosidase and sialidase in the absence (Fig. 1B) or presence of PNGaseF (Fig. 1C), followed by western blot analysis. Fig. 1B shows that treatment with a mixture of endo-O-glycosidase and sialidase reduced the apparent molecular weight of bands by∼5 kDa in both cell lysate and conditioned medium samples. The additive treatment with PNGaseF (Fig. 1C), removing N-linked glycosyl groups, generated in the lysate sample an ALCAM isoform corresponding to the full-length protein of 63 kDa, while in the conditioned medium two sALCAM isoform of ∼55 and 35 kDa were observed. This finding suggests that 96 and 60 kDa isoforms detected in conditioned medium result from alternative cleavages and related glycosylation patterns.

### Release of ALCAM is inducible by ionomycin and PMA

**Figure 2:**
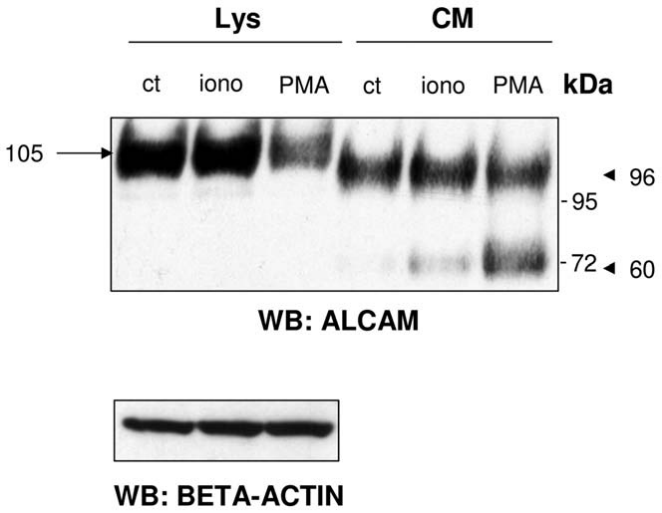
Changes in ALCAM expression induced by ionomycin or phorbol myristate acetate (PMA). Total lysates (Lys) and conditioned medium (CM) from TPC-1 cells, treated or not with 1 µM ionomycin (iono) or 50 ng/mL PMA for 2 h, were resolved by 3–8% SDS-PAGE and immunoblotted with ALCAM antibody. Both treatments led to an increase of secreted ALCAM isoforms; especially PMA induced a gain of 60-kDa isoform expression. Arrow indicates the membrane-localized ALCAM isoform in lysate, and arrowheads indicate shed-ALCAM isoforms in CM, respectively. Beta-actin was used as a loading control.

Protein shedding can be stimulated by calcium ionophores as ionomycin, [30] or by APMA (4-aminophenylmercuric acetate) [31] or PMA [28]. To investigate the regulation of ALCAM shedding, we treated the TPC-1 cell line with ionomycin and PMA. Treatment with either ionomycin or PMA increased the total level of soluble ALCAM isoforms (Fig. 2). Intriguingly, the treatment with PMA increased the expression of the 60-kDa ALCAM isoform, suggesting that this previously undescribed soluble form is produced by an alternative and inducible shedding of the 105-kDa ALCAM isoform.

### Release of ALCAM is sensitive to ADAM17/TACE inhibition

**Figure 3:**
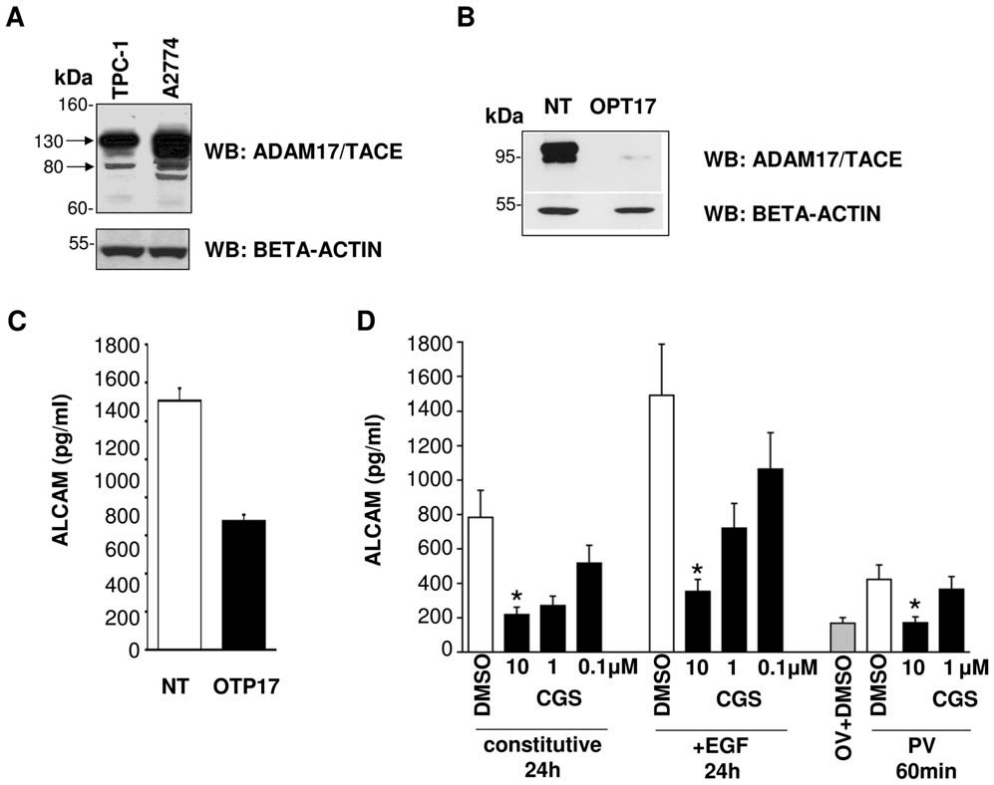
Release of ALCAM is sensitive to ADAM17/TACE silencing. (A) Analysis of lysates from TPC-1 and A2774 cells, resolved on 4–12% SDS-PAGE and immunoblotted with anti-ADAM17/TACE antibodies. Arrows indicate inactive (130 kDa) and active (80 kDa) forms of ADAM17/TACE enzyme. Normalization of results was obtained with immunoblotting analysis of beta-actin. (B) Expression of ADAM17/TACE protein by TPC-1 cells transfected with an ADAM17/TACE-specific small interfering RNA (siRNA) (OTP17), or with non-targeting siRNA (NT) as detected by western blot. The amount of protein was calculated by comparative densitometric scanning with beta-actin. (C) ELISA detection of ALCAM release by TPC-1 cells after transfection with siRNA specifically inhibiting ADAM-17/TACE (OTP17, black column) or with non-targeting siRNA (NT, white column). (D) Conditioned medium (CM) from TPC-1 cells, cultured with pervanadate (PV) (60 min), epidermal growth factor (EGF) (24 h), or medium alone (ctr, 24 h) in the presence (black columns) or in absence (white columns) of CGS27023A (CGS) was assessed by ELISA for ALCAM. Columns, means of three experiments (cells cultured in presence of 10, 1, or 0.1 µM CGS); bars, SD. *, P<0.05. Grey bar: in absence of CGS, but in presence of orthovanadate (OV).

We have previously demonstrated that EOC cells constitutively release ALCAM, and that cell treatment with EGF enhances this process, while ADAM17/TACE silencing inhibits it [28]. We then tested the effects of ADAM17/TACE inhibition and activation on ALCAM release in TPC-1 cells. TPC-1 cells indeed expressed ADAM17/TACE (Fig. 3A) and transfection of a specific siRNA targeting ADAM17/TACE significantly inhibited ADAM17/TACE protein expression (Fig. 3B) and sALCAM release (49% inhibition, Fig. 3C). We further determined whether epidermal growth factor receptor (EGFR) activation influenced sALCAM release via ADAM17/TACE in TPC-1 cells. To this end, cells were treated with PV or EGF in the presence of CGS (IC_50_ = 159±9 nmol/L on recombinant ADAM17/TACE *in vitro*, unpublished data) or solvent (DMSO). EGF increased ALCAM secretion by about 2-fold above spontaneous release at 24 h. Cell stimulation with PV also increased ALCAM release by two-fold within 60 min. In a dose-dependent manner, CGS inhibited ALCAM release induced by both stimuli and also by constitutive shedding (Fig. 3D), suggesting a role for ADAM17/TACE in ALCAM shedding in TPC-1 cells.

### ADAM17/TACE involvement in TPC-1 cell motility

We also examined TPC-1 cell motility using the scratch assay in presence of the ADAM17/TACE inhibitor CGS.

**Figure 4:**
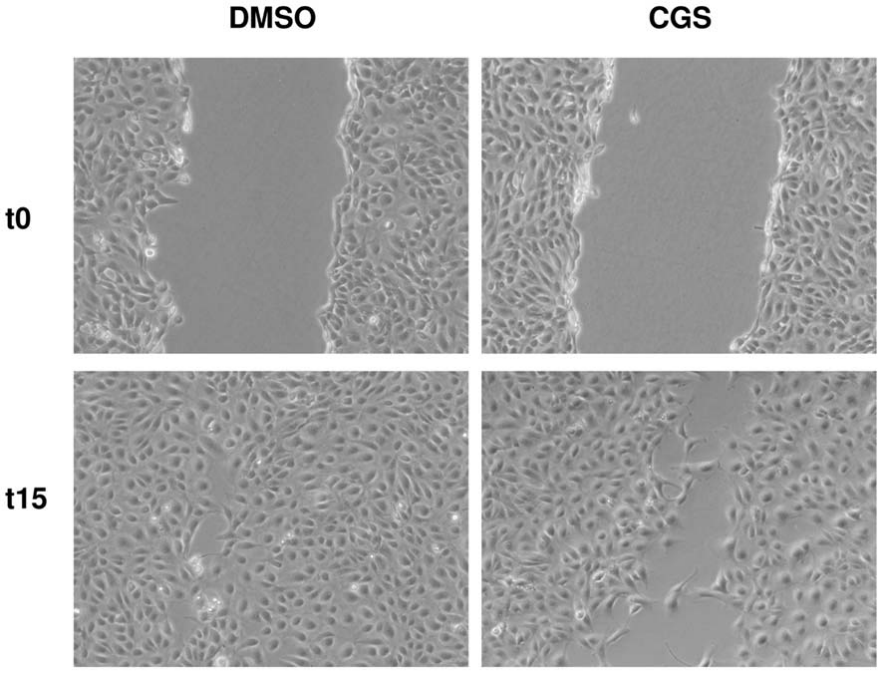
CGS decreases TPC-1 cell motility. Cells were seeded onto cell culture plates, as described in materials and methods. TPC-1 cells were exposed to CGS treatment or vehicle. At cell confluence, a scratch was performed using a 10 µl tip and the wound measured. After a 15 h treatment, the plate area remaining free of cells was measured. The cell-free area accounted for 44.5% of the image when the scratch was applied, P<0.05 versus DMSO control.

The analysis revealed that the distance covered by migrating cells was significantly decreased when TPC-1 cells were exposed to the CGS compared to cells exposed to the vehicle (DMSO) (Fig. 4). This result indicates a correlation between TPC-1 motile behavior and ADAM17/TACE activity.

Since the recombinant anti-ALCAM antibody I/F8 increased EOC cells motility in a scratch assay by altering ALCAM adhesive functions [28], we examined its activity on the TPC-1 cell line. However, the use of I/F8 antibody failed to inhibit adhesion to the substrate and migration through an 8 µm pore diameter membrane and motility in the scratch assay (data not shown). The possible reason for the efficacy of ADAM17/TACE inhibitors and for the lack of effect of ALCAM antibody blockade may relate to the ability of ADAM17/TACE to cleave, besides ALCAM, several membrane substrates, including other adhesion molecules of the CAM family [32], which may play a redundant role in TPC-1 cell adhesion and motility.

### ALCAM expression in normal and neoplastic thyroid tissues

**Figure 5:**
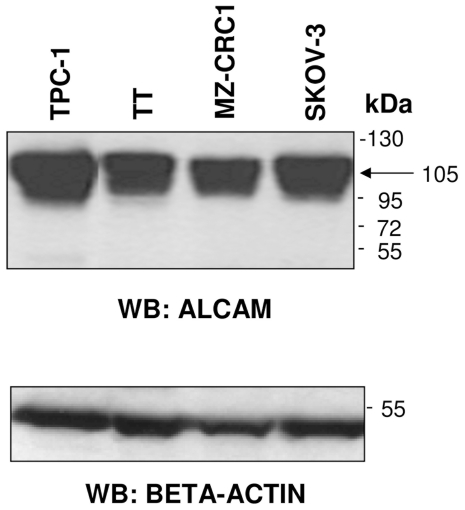
ALCAM expression in thyroid cell lines. Analysis of TT and MZ-CRC1 cell lysates showing ALCAM protein expression in these thyroid cell lines. SKOV-3 and TPC-1 cell lysates were included as controls. Total lysates from each cell line were resolved by 4–12% SDS-PAGE and immunoblotted with anti-ALCAM antibodies. Arrow indicates the fully glycosylated ALCAM isoform. Beta-actin was used as a loading control.

**Figure 6:**
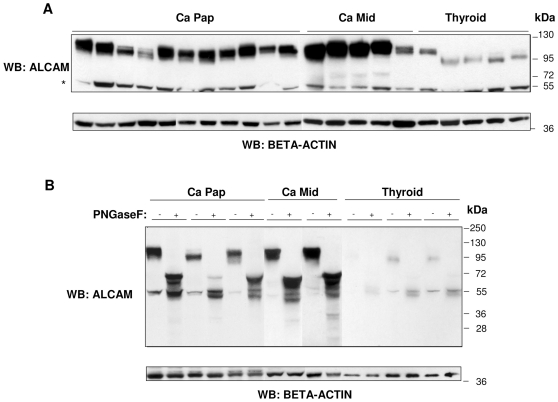
ALCAM expression in normal and tumor human thyroid tissue samples. (A) Total protein extracts (30 µg) from 11 papillary thyroid carcinomas (Ca Pap), 5 medullary thyroid carcinomas (Ca Mid), and 5 normal thyroid tissues (Thyroid) were resolved by 4–12% SDS-PAGE, transferred onto a nitrocellulose membrane, and immunoblotted with anti-ALCAM antibody. (*) indicates a non-specific background band. Beta-actin was used as a loading control. (B) Western blot analysis of ALCAM expression of total protein extracts (30 µg) from three PTCs, two MTCs, and three CTRLs, treated (+) or not (−) with N-glycosidase F. Beta-actin was used as a loading control.

Western blot analysis indicated a high expression of ALCAM in the TT and MZ-CRC1 cell lines (Fig. 5). We further explored its expression in archival tissue samples from 11 PTCs, 5 MTCs, and 5 normal thyroids (CTRLs). As shown in Fig. 6A, the ALCAM staining pattern appeared to be stronger in tumor samples, especially in MTCs, and revealed several discrete bands ranging from 120 to 100 kDa, probably due to a different level of glycosylation. In particular, in normal thyroid tissues, ALCAM staining was weaker and associated with a protein of ∼100 kDa.

To better clarify the nature of the ALCAM isoforms, we performed a deglycosylation of the lysates of three TPCs, two MTCs, and three normal thyroids with PNGaseF, removing N-linked sugars and leaving the protein backbone. ALCAM immunoblotting of the deglycosylated normal thyroid samples (Fig. 6B) showed a principal band corresponding to the precursor of 68 kDa. In contrast, in deglycosylated tumor samples, we detected multiple bands, probably the result of a partial deglycosylation or degradation of the putative membrane-associated 105-kDa isoform [28] and/or a concomitant presence of other unknown post-transduction modifications. These results confirmed that the multiple isoforms observed in tumor samples exhibited a prevalence associated with differential N-linked glycosylation (Fig. 6A and B).

**Figure 7:**
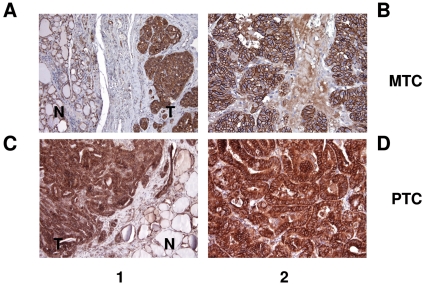
Immunohistochemical analysis of ALCAM. Membranous positive staining in surrounding non-tumor tissue (A and C) and in medullary thyroid carcinomas (MTCs) (B). Cytoplasmic and membranous positive staining in papillary thyroid carcinomas (PTCs) (D). 1, low-power field; 2, high-power field; N (normal tissue), T (tumor tissue).

To assess ALCAM expression and localization in normal and neoplastic thyroid tissues, we performed IHC analysis on formalin-fixed and paraffin-embedded tumoral sections of four PTCs and two MTCs. In comparison with peritumoral thyroid tissue, ALCAM was overexpressed in all PTC and MTC tissues. With regard to cellular localization, in MTC the surrounding non-tumor tissue (Fig. 7A) and the corresponding tumor tissue (Fig. 7B) showed membranous positive immunostaining, as in the surrounding non-tumor tissue of PTC (Fig. 7C); while in PTC tumor tissue ALCAM staining was localized at the membrane and in the cytoplasm (Fig. 7D). These results are in agreement with the annotated immunohistochemical patterns in Human Protein Atlas database (www.proteinatlas.org).

We also used IHC to investigate the expression of CD56 and TTF-1 in sequential sections. Intriguingly, we found that in three out of four tested PTCs, both proteins were lost. By contrast, two out of two tested MTCs showed positivity for CD56 and ALCAM and negativity for TTF-1 (Table 1).

**Table 1:** Immunohistochemical reactivity of thyroid tumors.

Case n.	ALCAM		TTF-1	CD56	Carcinoma diagnosis
	Cytoplasmic staining	Membranous staining	Nuclear staining	Membranous staining	
1	-	+	-	+	MTC
2	-	+	-	+	MTC
3	+	+	+	+	PTC
4	+	+	-	-	PTC
5	+	+	-	-	PTC
6	+	+	-	-	PTC

ALCAM, activated leukocyte cell adhesion molecule; PTC, papillary thyroid carcinoma;

MTC, medullary thyroid carcinoma.

CD56 and TTF-1 were negative in three out of four PTC cases. MTCs showed comparable positive immunostaining results for CD56 but were completely negative for TTF-1.

## Discussion

Over the past decade, alterations in expression of ALCAM have been reported in several human tumors and recently in renal carcinoma and in neuroblastoma primary tumors [33]. We show that ALCAM expression is also modulated in thyroid cancer. Membrane-associated ALCAM isoforms were overexpressed and highly glycosylated in tumors; in contrast, normal thyroid tissue expressed preferentially low glycosylated ALCAM isoforms. ALCAM was also strongly released in conditioned medium. The proteomic characterization of ALCAM isoforms in TPC-1 cells indicated that two soluble isoforms are released into conditioned medium; in addition, it suggested an unpredicted use of O-glycosylation sites, other than the known N-glycosylation, that could be important for both compartmentalization and secretion [34].

IHC confirmed that ALCAM expression was higher in tumors than in adjacent, normal peritumoral thyroid. With regard to cellular localization, ALCAM staining was mostly located in the cell membrane in normal thyroids and in MTCs, while in PTCs was membrane associated and cytoplasmic. This diversity in staining patterns could reflect tumor biology across histologies or grading. On the basis of these data, ALCAM overexpression in neoplastic thyroid tissues, analogously to other tumor types, could be implicated in tumor invasiveness and dedifferentiation processes. This concept is consistent with the loss of CD56 and TTF-1 expression in PTCs, associated with increased tumor invasiveness *in vivo* [10]; [35]. Multiple studies have examined ALCAM expression at the transcript and protein levels in tumors. There is an emerging consensus that low membrane level of ALCAM is a bad prognostic marker in breast [24] and ovarian cancer [20]. In this respect, the possibility that the high levels of ALCAM expression in thyroid tumors may indicate a reprogramming in transcriptional activity due the loss TTF-1 may deserve further investigation.

Regarding medullary and papillary thyroid tumors one of the possible mechanisms involved in ALCAM overexpression could be RET oncogenic activation. However, RET inhibition failed to alter ALCAM levels in TPC-1, suggesting that ALCAM overexpression is not dependent on RET activity and that other factors are likely to be involved (unpublished results).

Instead, we found that EGFR stimulation increases ALCAM secretion in part through an ADAM17/TACE-dependent pathway. We previously demonstrated the expression of EGFR in MTC cell lines and clinical biopsies [36]. In addition, Rodriguez-Antona et al. [37] have reported, based on a large set of MTC cases, that EGFR is significantly overexpressed in metastases compared with primary tumors. EGFR is also expressed in PTCs, and EGFR signaling may represent a molecular target in poorly differentiated thyroid carcinoma cells [38]. Our data indicate that activated EGFR also cooperates in thyroid cancer cell lines with the ADAM17/TACE sheddase in promoting ALCAM shedding and soluble ALCAM release. We previously demonstrated that ADAM17/TACE activity is involved in motility of ovarian carcinoma cells [28].

In the present report we show that inhibition of ADAM17/TACE function through chemical inhibitor also interfered with TPC-1 cell migration.

This is explained by the fact that ADAM17/TACE cleavage of transmembrane proteins such as ALCAM is an essential process controlling many biological functions including cell adhesion and migration. A similar anti-adhesive activity would be also expected by antibody-mediated blockade of ALCAM adhesive functions, which increased ovarian cancer cells motility in a previous report [28]. However, in the case of TPC-1 cells the anti-ALCAM antibody I/F8 failed to augment cell motility. The possible reason of this discrepancy may depend upon the presence of several adhesion molecules, which display redundant functions in cell-cell adhesion and are all substrates of ADAM17/TACE. For example, a previous report showed the expression of ICAM-1 in thyroid carcinoma cells [39].

In parallel studies we observed that TPC-1 cells express a strong mesenchymal phenotype, as evidenced by the expression of mesenchymal cytoskeletal proteins, such as vimentin, and the high deposition of extracellular matrix proteins, including collagens and fibronectin [40]. Consistently with these observations, TPC-1 cells showed elevated levels of β1-integrin and integrin-regulated signaling pathways, which regulate intracellular signaling in control of survival, cell-matrix adhesion dynamics and cell migration [40].

Proteolysis of ALCAM in our study generated two sALCAM fragments of different molecular weights, which seemed not derived from differential glycosylation, as treatment with N- and O-glycosydases still yielded two molecular forms. A possible alternative hypothesis is that ALCAM can be cleaved at two distinct sites: one close to the membrane and another in a more membrane-distal region, which yield the 96-kDa and the 60-kDa glycosylated fragments, respectively. Similarly, digestion of immunoprecipitated ALCAM in vitro by recombinant ADAM17/TACE produced a fragment of about 60 kDa, suggesting that the same enzyme may cleave ALCAM in two different sites, in different conditions [33]. PMA stimulation of TPC-1 cells activated ADAM17/TACE activity as detected by the increased release of both 96 and the 60 kDa ALCAM isoforms, supporting a role of protein kinase C (PKC) in regulating ALCAM dynamics [41].

Future exploration of ALCAM differential function and regulation in thyroid epithelium cancer and relative metastasis will contribute to a better understanding of whether its dysregulation contributes to disease progression and may provide insight into whether ALCAM has active function in thyroid tumors.

## Materials and Methods

### Ethics Statements

This study, involving human subjects, has obtained ethics approval from Independent Ethics Committee (IEC) of Fondazione IRCCS Istituto Nazionale dei Tumori, Milan, Italy.

### Reagents, cell culture media, antibodies, and enzymes

Sodium orthovanadate (Na_3_VO_4_), PMA, ionomycin, PMSF, and DMSO were supplied from Sigma-Aldrich (Milan, Italy); protease inhibitor cocktail tablets (Complete Mini) from Roche (Milan, Italy); and Bradford reagent from BioRad (Milan, Italy). DMEM was from Gibco (Invitrogen; Milan, Italy); FBS and FCS from HyClone (Celbio; Milan, Italy); RPMI 1640, Ham's F12, sodium pyruvate, L-glutamine, and trypsin-EDTA solution from BioWhittaker (Lonza; Milan, Italy); and HEPES buffer from EuroClone (Celbio; Milan, Italy). Anti-ALCAM antibody was from Novocastra (Leica; Milan, Italy); anti–β-actin and anti-ADAM17/TACE antibodies were respectively from Sigma-Aldrich and AbCam (Cambridge, MA, USA). I/F8 scFv antibody is described in [42]. Anti–TTF-1 antibody was from Novus Biological (DBA, Milan, Italy). Anti-rabbit IgG, horseradish peroxidase–linked whole antibody (from donkey), anti-mouse IgG, and horseradish peroxidase–linked whole antibody (from sheep) were supplied from GE Healthcare (Milan, Italy); and anti-goat antiserum was from ZYMED Laboratories (Invitrogen; Milan, Italy). N-glycosidase F (PNGaseF) was purchased from New England Biolabs (Celbio; Milan, Italy) and O-glycosidase and α(2^→3,6,8,9)^neuraminidase (sialidase) from Sigma-Aldrich.

### Tissue samples and protein extraction

MTC and PTC specimens and normal thyroid tissues were obtained from 22 patients, who provided written informed consent to participate in the study according to the ethical guidelines of our Institute, after surgery. Tissue samples were pulverized and processed as described in [36].

### Preparation of cell lysates

Cells were lysed and processed as described in [36].

### Cell cultures

Cell lines were grown as follows: human PTC cell line TPC-1 in DMEM, supplemented with 10% FBS; human MTC cell line TT in Ham's F12, supplemented with 15% FCS; human MTC cell line MZ-CRC1 in DMEM, supplemented with 10% FCS; human ovarian cancer cell line A2774 [28] and human ovarian cancer cell line SKOV-3 (from American Type Culture Collection; Milan, Italy) in RPMI 1640, supplemented with 10% FCS.

### Preparation of conditioned media

TPC-1 cells were seeded at 3×10^4^ cells/cm^2^ and cultured for 4 d. Medium was removed, and cells were washed four times with PBS and once with serum-free medium. Serum-free DMEM was added and plates incubated for 24 h. The CM was collected, centrifuged at 4000 rpm for 10 min to remove cell debris, and finally concentrated and desalted using a spin column (Agilent Technologies; Milan, Italy).

### N-deglycosylation and O-deglycosylation reactions

Cell lysates and CM proteins were O-deglycosylated with a mixture of O-glycosidase and α(2→3,6,8,9) neuraminidase (sialidase), with or without PNGaseF. A total of 100 µg of protein from each sample was treated with 5 mU of O-glycosidase and 10 mU of sialidase. An additive treatment with PNGaseF was performed, using 1 U/100 µg of protein.

### SDS-PAGE and western blotting

The procedures were performed as previously described in [36].

### Drug treatments

To test effects of ionomycin or PMA on ALCAM secretion and shedding, TPC-1 cells were seeded at 3×10^4^ cells/cm^2^, grown for 3 d, and then treated with 1 µM or 50 ng/mL for 2 h, respectively.

### ADAM17/TACE inhibition and ELISA assay

ADAM 17/TACE was inhibited with metalloproteinase inhibitor CGS27023A (CGS) as previously described [28] Conditioned media from TPC-1 and A2774 cells treated with PV, EGF, or medium alone with or without CGS were then collected, centrifuged at 1000 rpm, and used undiluted for secreted ALCAM detection with the DuoSet ELISA Development Kit (R&D Systems; Milan, Italy) based on the anti-human ALCAM mAB Clone 105902 as capturer and on a biotinylated polyclonal goat anti-human ALCAM serum as the detection antibody. Assays were done in triplicate, and background values were subtracted. Data were expressed as the mean ± SD and analyzed using a two-tailed Student's *t* test or the Mann-Whitney test.

### Small interfering RNA (siRNA) transfection

TPC-1 cells were transfected with the ON-TARGET plus SMART pool for human ADAM17/TACE or siCONTROL Non-Targeting siRNA pool (Dharmacon; Milan, Italy), using Lipofectamine RNAiMAX (Invitrogen). Transfected cultures were assayed for constitutive or induced ALCAM shedding 48 h after siRNA delivery. Cells were harvested by PBS-EDTA treatment and processed for RNA and protein extraction to detect ADAM17/TACE mRNA and protein.

### Scratch assay

The assay was made as previously described [28] and visualized by comparing photographs taken at the time of addition of EGF and 15 h later, by a Nikon DS-5M Camera System mounted on a phase-contrast Leitz microscope. The distance traveled by the cells was determined by measuring the cell-free area width at time 15 h and subtracting it from the cell-free area width at time 0. The values obtained were then expressed as % migration, setting the gap width at *t*_0_ as 100%. Two experiments were done in triplicates.

### Immunohistochemistry (IHC)

The IHC analyses were made using 2 µm of the formalin-fixed and paraffin-embedded tumoral sections with antibodies against ALCAM and CD56 as described in Mezzanzanica et al. [20] and in Scarpino et al., [10] respectively. All of the samples were also immunophenotyped using an anti–TTF-1 antibody (diluted 1∶75), and the antigens were retrieved using 10 mM citrate buffer (pH 6.0) in an autoclave at 95°C for 15 min.
